# Trait analysis reveals DOG1 determines initial depth of seed dormancy, but not changes during dormancy cycling that result in seedling emergence timing

**DOI:** 10.1111/nph.16081

**Published:** 2019-09-18

**Authors:** Steven Footitt, Peter G. Walley, James R. Lynn, Angela J. Hambidge, Steven Penfield, William E. Finch‐Savage

**Affiliations:** ^1^ School of Life Sciences University of Warwick Wellesbourne Campus Warwickshire CV35 9EF UK; ^2^ Functional and Comparative Genomics Institute of Integrative Biology University of Liverpool Liverpool L69 7ZB UK; ^3^ Applied Statistical Solutions Bishops Tachbrook Leamington CV33 9RJ UK; ^4^ Department of Crop Genetics John Innes Centre Norwich Research Park Norwich NR4 7UH UK

**Keywords:** ABA signalling, Arabidopsis, DOG1, dormancy cycling, germination, QTL analysis, seed dormancy

## Abstract

- Seedling emergence timing is crucial in competitive plant communities and so contributes to species fitness. To understand the mechanistic basis of variation in seedling emergence timing, we exploited the contrasting behaviour of two *Arabidopsis thaliana* ecotypes: Cape Verde Islands (Cvi) and Burren (Bur‐0).
- We used RNA‐Seq analysis of RNA from exhumed seeds and quantitative trait loci (QTL) analyses on a mapping population from crossing the Cvi and Bur‐0 ecotypes.
- We determined genome‐wide expression patterns over an annual dormancy cycle in both ecotypes, identifying nine major clusters based on the seasonal timing of gene expression, and variation in behaviour between them. QTL were identified for depth of seed dormancy and seedling emergence timing (SET).
- Both analyses showed a key role for *DOG1* in determining depth of dormancy, but did not support a direct role for *DOG1* in generating altered seasonal patterns of seedling emergence. The principle QTL determining SET (*SET1*: dormancy cycling) is physically close on chromosome 5, but is distinct from *DOG1*. We show that *SET1* and two other *SET* QTLs each contain a candidate gene (*AHG1*,* ANAC060*,* PDF1* respectively) closely associated with *DOG1* and abscisic acid signalling and suggest a model for the control of SET in the field.

## Abstract 2

See also the Commentary on this article by https://doi.org/10.1111/nph.16254.

## Introduction

Annual plant seeds are most often shed in a dormant state. The depth of this dormancy differs between species and is greatly influenced by the maternal environment. However, once shed the depth of dormancy adjusts to the seedbank environment and continually changes in an annual cycle (Finch‐Savage & Leubner‐Metzger, 2006). It is this process that ensures that germination and subsequent seedling growth takes place in a favourable habitat and climate space during the correct season for the resulting plant to complete its life cycle. Dormancy cycling is therefore essential to species fitness and the competitiveness of weeds in crop production practice. Understanding this process is crucial to the future management of natural plant populations and the development of more environmentally benign cultural weed management practices.

Many molecular mechanisms that can regulate dormancy have been identified individually in controlled laboratory studies (Finch‐Savage & Leubner‐Metzger, 2006; Holdsworth *et al*., 2008; North *et al*., 2010; Graeber *et al*., 2012; El‐Maarouf‐Bouteau *et al*., 2013; Dekkers & Bentsink, 2015; Rodríguez *et al*., 2015; Nee *et al*., 2017; Nishimura *et al*., 2018). However, our understanding of how the seed employs this complex suite of mechanisms during dormancy cycling in response to the variable environment (principally in terms of temperature) of the soil seed bank is only just developing. The essential feature of dormancy cycles is that seeds remain dormant and non‐germinating throughout this process, but sensitivity (depth of dormancy) to spatial factors in the environment (principally light) changes (Finch‐Savage & Footitt, 2012). Seeds only progress to germination completion on exposure to these factors when seeds have become sensitive to them, and it is this process that determines germination and seedling emergence timing (Finch‐Savage & Footitt, 2017).

A model for the regulation of dormancy cycling in *Arabidopsis thaliana* has been proposed (Finch‐Savage & Footitt, 2017). Central to this is the hormone balance between gibberellins (GA) and abscisic acid (ABA) through both synthesis and sensitivity. Environmental signals such as light and nitrate feed directly into that balance via up‐regulating *GIBBERELLIC ACID 3‐OXIADASE 1* (GA3ox1; GA synthesis) and CYTOCHROME P450 707A2 (CYP707A2; ABA catabolism) expression, respectively, to favour loss of dormancy and germination completion when dormancy is shallow (Finch‐Savage & Footitt, 2017). Overriding this is the response to temperature, which drives seasonal changes in the level of expression of many genes linked to the hormone balance (Cadman *et al*., 2006; Finch‐Savage *et al*., 2007), but also DOG1 (DELAY OF GERMINATION 1; Bentsink *et al*., 2006), a putative DNA‐binding transcription factor that is linked to the accumulation of thermal time (Footitt *et al*., 2015). These determine the depth of dormancy as the seasons change in the annual cycle (Footitt *et al*., 2011, 2013, 2014). DOG1 is essential and its mutation can completely remove seed dormancy (Bentsink *et al*., 2006). Understanding of the key factors linking DOG1 to the hormone balance and regulation of dormancy is now developing. DOG1 has been shown to physically interact with two phosphatases (ABA‐HYPERSENSITIVE GERMINATION 1 and 3; AHG1 and 3) to functionally block their essential downstream roles in the release of seed dormancy (Nee *et al*., 2017). A further phosphatase, PROTEIN PHOSPHATASE 2A SUBUNIT A2 (*PP2AA/PDF1*)), also physically interacts with DOG1 but acts upstream to have a negative role in seed dormancy. These phosphatases are a potential link between DOG1 and the regulation of seasonal patterns in the depth of dormancy.

DOG1 transduces environmental effects during maturation to alter depth of dormancy (Kendall *et al*., 2011; Nakabayashi *et al*., 2012), and subsequent changes at the chromatin level are closely linked to environmental signals in the soil seedbank that determine changes in the depth of dormancy (Footitt *et al*., 2015). The latter was suggested as a means by which to accumulate thermal time and influence the timing of germination through seed dormancy cycling, thus linking DOG1 and dormancy cycling. Further support for this view comes from two studies by Huang *et al*. (2010) and Postma & Agren (2016), who suggest that *DOG1* is the principal quantitative trait locus (QTL) affecting the pattern of seedling establishment in the field. However, the annual pattern of *DOG1* expression is correlated to the seasonal temperature pattern in different ecotypes (Cvi and Bur‐0), even though they have contrasting seed dormancy cycles. Thus *DOG1* expression follows environmental cues and does not appear to directly determine the pattern of dormancy cycling (Footitt *et al*., 2013). It therefore remains unclear whether DOG1 variation itself can drive variation in dormancy cycling behaviour.

To develop a better understanding of dormancy cycling, we used the Arabidopsis ecotypes Cape Verde Islands (Cvi) and Burren (Bur‐0). These ecotypes are adapted to warm/dry and cool/damp climates respectively. As a result they have contrasting obligate winter and summer annual behaviour in the experimental environment used (Footitt *et al*., 2013). We exploited this adaptation to diverse climates to compare full genome expression patterns across annual dormancy cycles. Furthermore, to dissect the control of this downstream expression we constructed a new recombinant inbred line (RIL) mapping population from these same ecotypes. Using this population we screened for depth of dormancy in controlled laboratory conditions. We then screened for timing of seedling emergence (annual dormancy cycle) in a field‐based thermogradient tunnel under simulated global warming scenarios. Analysis of data from these controlled environments shows *DOG1* to be the principal QTL for depth of seed dormancy in mature seeds. However, analysis of data following sowing in variable environments shows that the principle QTL determining subsequent seedling emergence patterns (dormancy cycling; *SET1*) is physically close on chromosome 5, but is distinct from *DOG1*. Furthermore, we show that QTLs for emergence time contain genes closely associated to DOG1 signal transduction and ABA signalling pathways and discuss a model for the control of seedling emergence timing in the field.

## Materials and Methods

The *Arabidopsis thaliana* (L.) Heynh ecotypes Cape Verde Islands (Cvi; N8580) and Burren (Bur‐0; CS6643) were used in a series of experiments. Seed dormancy cycles were carried out in the field to determine genome‐wide gene expression patterns, and an RIL mapping population was developed between these obligate winter (Cvi) and summer annual (Bur) ecotypes. The population was used to screen depth of seed dormancy at maturity in controlled environments, and seedling emergence was recorded in a range of naturally variable environments. The data collected were subjected to QTL analyses.

### Seed production and gene expression during dormancy cycling in field soils

Seed production and experimental procedures for their dormancy cycling in field soil were as described elsewhere (Footitt *et al*., 2011, 2013). Total RNA was extracted from 50 mg seeds recovered from field soils during dormancy cycling as described previously (Footitt *et al*., 2013) for genome‐wide gene expression analysis using RNA‐Seq. Before burial, seed samples were taken from Arabidopsis Bur‐0 and Cvi for analysis (time zero). Seeds were buried in October 2009 and exhumed monthly to August 2010 (giving 11 time points) in Bur‐0. In Cvi seeds were buried in October 2007 and exhumed monthly to September 2008 (giving 12 time points). Sequencing was then carried out using a HiSeq 2000 (Illumina, San Diego, CA, USA) device. Between 64 million and 87 million 101‐bp paired‐end reads from Illumina libraries were prepared from the F3 bulked samples, respectively, and were aligned against the Arabidopsis Col‐0 TAIR10 reference sequence using tophat v.1.4.1 (https://cole-trapnell-lab.github.io/projects/tophat/) to the Arabidopsis TAIR10 reference sequence. The data can be found in the European Nucleotide Archive, accession no. PRJEB33535. Only samples that passed quality control were considered; for January in Bur‐0 and August in Cvi, only one sample passed quality control – others were excluded from the analysis. The cufflinks package was used to quantify gene and isoform abundance and quantify gene and isoform differential expression (Trapnell *et al*., 2012). To understand major seasonal gene expression, genes significantly differentially regulated between at least two time points were calculated using cuffdiff and a false discovery rate of 5%. Genes with a minimum fold change of at least 4‐fold were used for K‐means clustering, varying the value of K to optimize cluster composition to contain genes with similar gene expression patterns.

### Cvi × Bur‐0 mapping population

The Cvi (maternal line) ecotype was crossed with Bur‐0 (paternal line). The resulting F_1_ seeds were dry after‐ripened at room temperature for 2 months. Seeds (F_2_) from one F_1_ plant were used for single seed descent to the F_8_ generation under glasshouse conditions. Measures to prevent biasing the population for low dormancy were adapted from Alonso‐Blanco *et al*. (1998). 184 F_8_ lines were selected for production of the F_9_ generation in growth cabinets at two temperatures (15°C and 21°C under a 16 h : 8 h, light : dark cycle at a light intensity of 100 μmol m^−2^ s^−1^ and 80% relative humidity (gradually reduced to 45%)) to produce populations with different degrees of dormancy. Full details of population production and genotyping are provided in Supporting Information Methods S1.

### Screening for seed dormancy in the F_9_ generation

Germination was tested at 10 and 20°C in the light to evaluate the degree of low and high temperature thermo‐dormancy. In addition, seeds produced at 15°C were incubated at 20°C in the presence of 10 mM KNO_3_ to evaluate nitrate sensitivity, and were dry after‐ripened at 20°C in the dark for 30 d, then returned to −80°C before testing germination at 20°C. This, by comparison with germination at 20°C before after‐ripening, indicated the depth of dormancy in each line. Each test was repeated to give three independent replicates. Germination recording and analysis used the GERMINATOR system as described in Joosen *et al*. (2010;, see Methods S1). Final percentage germination data were analysed in genstat (VSN International, 2013) using the REML algorithm, following an empirical logit transformation, in which the data were adjusted by 0.5% to move germination rates away from 0 and 100%. Replicate tests, box within tests, and a residual were taken as random factors. Means from this analysis were subjected to QTL analysis.

### Seedling emergence of F_9_ seeds under global warming scenarios in a thermogradient tunnel

A projected median emissions scenario for the local experimental area used in this work (West Midlands, UK) indicates an increase in the summer mean temperature of 3.7°C by 2080, compared to the recent past (1981–2000; UK Climate Change Projections, 2014; http://ukclimateprojections.metoffice.gov.uk/). We used a thermogradient tunnel (Wurr *et al*., 1996) to establish a gradient from ambient to *c*. + 4°C, which gave a soil temperature gradient of 2.5°C. Seedling emergence was recorded at three positions along the gradient (termed ‘ambient’, ‘middle’ and ‘warm’) on 86 RILs chosen to represent the full range of characteristics seen in the population. The experiment was repeated on two occasions to simulate seed dispersal in the spring on 15–16 May 2013 (winter annual behaviour) and in the autumn on 22–23 October 2013 (summer annual behaviour). Full details of the procedures used in these emergence experiments are provided in Methods S1. To quantify the tendency to behave as a winter annual, the parameter ‘emerge’ was calculated, for each of the RILs, as the percentage of seedlings which emerged during the periods after the mean soil temperatures had reached the annual maximum, and before they had reached the annual minimum. These periods were the same for each of the three regions within the thermogradient tunnel. Data from the spring and autumn set‐up times were combined.

### QTL analysis

Quantitative trait loci analyses were performed according to Walley *et al*. (2012). Briefly, mean RIL trait data were used as input for QTL analysis using mapQTL^®^ 6 (Van Ooijen, 2009), and R/qtl v1.39‐5 (Broman *et al*., 2003) in R v.3.3.0 (R Core Team, 2016) for comparisons. Interval mapping (single‐QTL model) was first implemented in mapQTL^®^ 6, and results were compared to results from the ‘scanone’ function with the ‘EM’ algorithm in R/qtl; empirical genome‐wide LOD significance thresholds were determined using permutation tests (1000 iterations), and QTL was declared when α ≤ 0.05. Markers linked to QTL were then used as cofactors in approximate multiple QTL models (MQM), as implemented in mapQTL^®^ 6. QTL models were recalculated using different combinations of cofactors in a stepwise approach until there was no change in the recorded LOD and *R* ^2^ associated with the QTL model. Final QTL coordinates were delimited using 1.0 and 1.5 LOD support intervals. QTL coordinates were used to illustrate QTL locations on the linkage map using mapchart (Voorrips, 2002).

## Results

**Figure 1:**
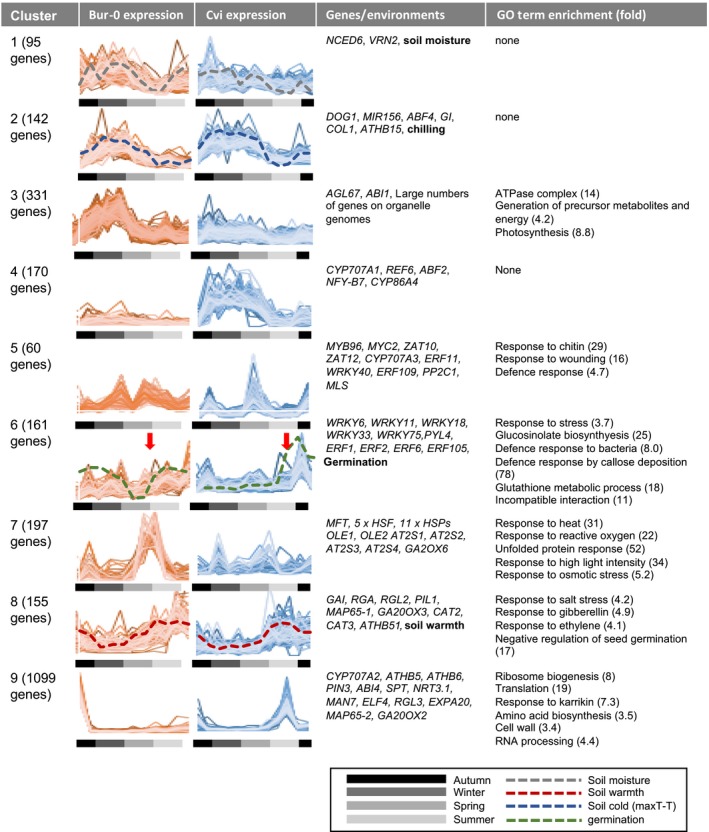
Gene expression patterns for *Arabidopsis thaliana* ecotypes Cape Verde Islands (Cvi) and Burren (Bur‐0) during an annual dormancy cycle; cluster analysis of RNA‐Seq data from samples exhumed at intervals over 11‐month (Cvi) and 10‐month (Bur‐0) periods following sowing in October. Seasons are indicated along with example genes and a summary of gene ontology term analysis in each cluster. Soil warmth (red dotted) shows the pattern of maximum temperature and soil cold (Maximum recorded temperature – actual temperature (maxT–T)) shows an inverted pattern of minimum temperature (blue dotted). Soil moisture (grey dotted) is also shown. Potential to germinate on exhumation and exposure to light (i.e. pattern of dormancy; green dotted) is shown with the time of seedling emergence in disturbed plots (red arrows). Environmental parameters and germination rates were published previously (Footitt *et al*., 2011, 2013).

To understand the mechanistic basis of variation in seedling emergence timing, we compared and exploited the contrasting behaviour of two Arabidopsis ecotypes: Cape Verde Islands (Cvi) and Burren (Bur‐0). In the local climate of the experimental area, Cvi and Bur‐0 have been shown to be obligate winter and summer annuals respectively (Footitt *et al*., 2013; Fig. 1).

### Analysis of genome‐wide expression patterns over an annual seasonal cycle

We sowed seeds in fully‐replicated randomized block field experiments in 2007 and 2009 and then exhumed seeds at monthly intervals over 1 yr. Throughout, seeds remained below ground until exhumed in the dark and were therefore dormant in the absence of exposure to light. RNA‐Seq analysis of total RNA from exhumed seeds enabled the determination of differences in genome‐wide expression patterns over an annual seasonal cycle between the two ecotypes using Illumina short read sequencing (see the Materials and Methods section; Fig. 1, Table S1). Potential for germination (depth of dormancy) was determined by exposing these seeds to light. Seeds of the two ecotypes had contrasting patterns in depth of dormancy; as a result germination potential increased at the times of year characteristic of winter annuals (Cvi) and summer annuals (Bur‐0) (late summer and late spring respectively; Footitt *et al*., 2013; Fig. 1). To understand the principle differences in gene expression patterns between the ecotypes we used K‐means cluster analysis, progressively decreasing the number of clusters until all remaining clusters showed distinct gene expression patterns. This identified nine major clusters based on the seasonal timing of gene expression and variation in behaviour between Bur‐0 and Cvi. To understand the significance of each cluster, we used gene ontology (GO) term analysis and additionally identified known transcripts with described roles in seed dormancy or germination within each cluster (Table S1). Addition of environmental variables into the cluster analysis enabled their association with major gene expression patterns (Fig. 1). Distinct clusters of annual gene expression patterns were revealed. Three clusters (clusters 1, 2 and 8) were associated with environmental variables and were common to both ecotypes. In line with their different patterns of depth of dormancy, the ecotypes also exhibited different and sometimes contrasting (clusters 3, 4, 5, 6, 7 and 9) gene expression profiles, indicating temporal separation of gene expression driven by climate adaptation. Each cluster contained genes linked to seed dormancy and germination as summarized in Fig. 1, and full groupings of genes in each cluster are shown in Table S1.

Clusters 1–4 contained genes that were expressed predominantly in winter, associated with either high soil moisture or low temperature, in both ecotypes (clusters 1 and 2) or unique to Bur‐0 or Cvi (clusters 3 and 4). This included *DOG1*,* NCED6* (*NINE‐CIS‐EPOXYCAROTENOID DIOXYGENASE 6*), *MIR156* (MicroRNA156) and *ABI1* (*ABA INSENSITIVE 1*), all of which have previously been linked to deep dormancy. Counterbalancing this is a major summer‐expressed cluster (cluster 8) common to Cvi and Bur‐0 that contains genes expressed in shallow dormant seeds, such as genes encoding the DELLA proteins and GIBBERELLIN 20‐OXIDASE (GA20OX).

Four other major clusters had altered expression dynamics between the two ecotypes. Cluster 5 contains genes that are specifically down‐regulated in April in Bur‐0, coinciding with the annual increase in seed dormancy. Interestingly these same genes were rapidly induced in Cvi at the same time of year as dormancy was declining. Thus, cluster 5 is alternatively regulated between Bur‐0 and Cvi. It is enriched in genes associated with wounding and defence responses (*P* < 0.001) and includes transcription factors that confer the ABA response (*MYC2*,* MYB‐RELATED PROTEIN 96* (*MYB96*)).

Related to cluster 5 was cluster 6, which showed a similar gene expression pattern in Bur‐0, but in Cvi this cluster was expressed during the summer seedling emergence window. This cluster contains genes whose expression is positively correlated with the shallow dormant state in both ecotypes and coincides with germination/seedling emergence recorded in separate plots that were regularly disturbed to expose seeds to light (Fig. 1; time of emergence indicated by red arrows). These ‘shallow dormancy’ genes may be associated with fine‐tuning of germination timing, in contrast to the ‘winter genes’ that are likely linked by the annual temperature cycle to deep dormancy and prevention of germination in winter in both ecotypes. Cluster 6 contained genes that are targets of the N‐end rule pathway in seeds, such as group VII ERF transcription factors (Vicente *et al*., 2017, 2019), and WRKY transcription factors associated with defence responses (Jiang *et al*., 2017). The analysis suggests that the regulation of clusters 5 and 6 was related to ‘shallow’ dormancy control (i.e. after loss of deep dormancy in Cvi and change in depth of dormancy in the shallow dormant Bur‐0). These clusters were dominated by oxylipin, defence, ethylene and ABA‐associated response genes.

Cluster 7 is dominated by genes associated with the maturation programme, including seed storage proteins, oleosins and heat shock transcription factors (HSFs), along with the dormancy‐regulator *MOTHER OF FT AND TFL1* (*MFT*) (Dave *et al*., 2016). Their expression was not related to temperature but instead peaked before, or during, dormancy loss. Peak expression therefore occurs at the point at which seeds have an increasing potential to germinate and are therefore susceptible to environmental stresses. Genes in this cluster may therefore be reprising their role in seed maturation when desiccation tolerance is established. Finally, cluster 9 contains 1099 genes strongly expressed in germinating seeds, such as ribosomal proteins, *SPATULA*, cell wall remodelling genes and *CYP707A2* (*ABSCISIC ACID 8′‐HYDROXYLASE 2*). These were highly expressed in mature Bur‐0 seeds at burial, but only expressed in Cvi during the summer emergence window (cluster 9). This cluster also contains gibberellin oxidases such as the *GA20OX*s and a *GA2OX* (*GA2OX6* known to be cold induced), but no *GA3OX*s, which are linked to the completion of germination. These genes are highly expressed in germinating seeds, compared to dormant seeds (Finch‐Savage *et al*., 2007), which reflects the fact that Bur‐0 seeds are capable of immediate germination if given light (Footitt *et al*., 2013).

**Figure 2:**
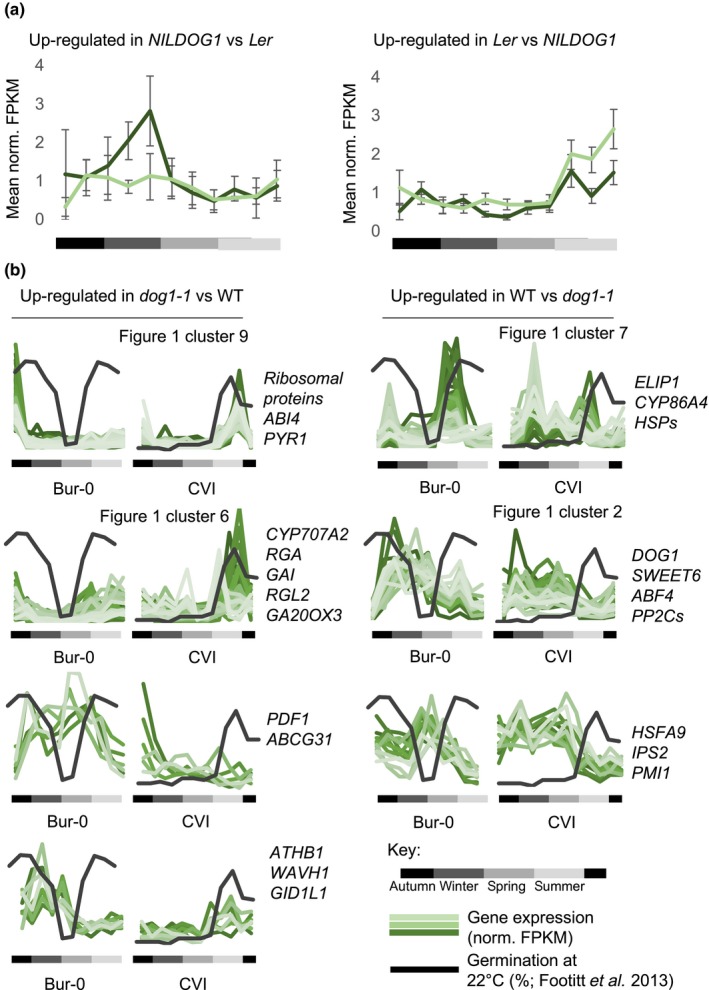
Annual expression patterns of DOG1 influenced genes in *Arabidopsis thaliana*. (a) Annual expression pattern of genes up‐ and down‐regulated in *NILDOG1* compared to *Ler* identified in Bentsink *et al*. (2010); dark green line is Cape Verde Islands (Cvi) and light green line is Burren (Bur‐0). (b) Cluster analysis of the annual expression patterns of genes up‐ and down‐regulated in *dog1‐1* compared to the wild‐type identified by Dekkers *et al*. (2013). Names of selected genes are alongside each cluster, and the changing level of germination of exhumed seeds at 20°C in the light indicates the depth of dormancy. Error bars indicate ± SE. norm. FPKB, normalized fragments per kilobase; WT, wild‐type.

Because DOG1 has been implicated in control of field emergence timing, we took DOG1‐dependent gene expression (Bentsink *et al*., 2010; Dekkers *et al*., 2013), and assessed how these genes behaved in buried Bur‐0 and Cvi seeds (Fig. 2). Using near‐isogenic lines (NILs), Bentsink *et al*. (2010) identified DOG1‐up‐regulated genes by comparing gene expression in seeds carrying the strong Cvi allele (NIL‐DOG1) and the weak *Ler* *DOG1* allele. In our RNA‐Seq data those *DOG1* ^CVI^‐up‐regulated genes were expressed in winter at a higher level in Cvi than in Bur‐0 (Fig. 2a). By contrast, genes up‐regulated in the *DOG1* ^LER^ background were expressed in summer and were higher in Bur‐0. This shows that DOG1 affects gene expression year‐round in buried seeds. In the Dekkers *et al*. (2013) dataset (Fig. 2b), genes highly expressed in *dog1‐1* compared to wild‐type were primarily those in the germination‐associated clusters 6 and 9 shown in Fig. 1 (see also Table S1), suggesting that these genes are those that, in general, are highly expressed in less dormant seeds. Focussing on genes whose expression in seeds depends on DOG1, a major class of these were in cluster 7 (maturation‐associated) or cluster 2, which are expressed, along with *DOG1* itself, in winter (Fig. 1). In CVI these maturation‐associated genes are expressed predominantly in the secondary dormant phase (Fig. 1), but in Bur‐0 they are highly expressed on exit from secondary dormancy. However, there were no DOG1‐regulated genes in cluster 5, which contains the gene expression profiles most strongly associated with differences in germination timing between Bur‐0 and Cvi. Overall, our analysis shows a key role for *DOG1* in determining the depth of primary dormancy, in the regulation of genes co‐expressed with DOG1 in winter, and in coupling the high expression of maturation‐associated genes to the dormant phase. However, we found no evidence that DOG1 generally controls the expression of genes which show altered seasons of expression between Bur‐0 and Cvi (Fig. 1 cluster 5), suggesting that these are affected by a DOG1‐independent mechanism.

### Genetic control of depth of dormancy and field emergence time

**Figure 3:**
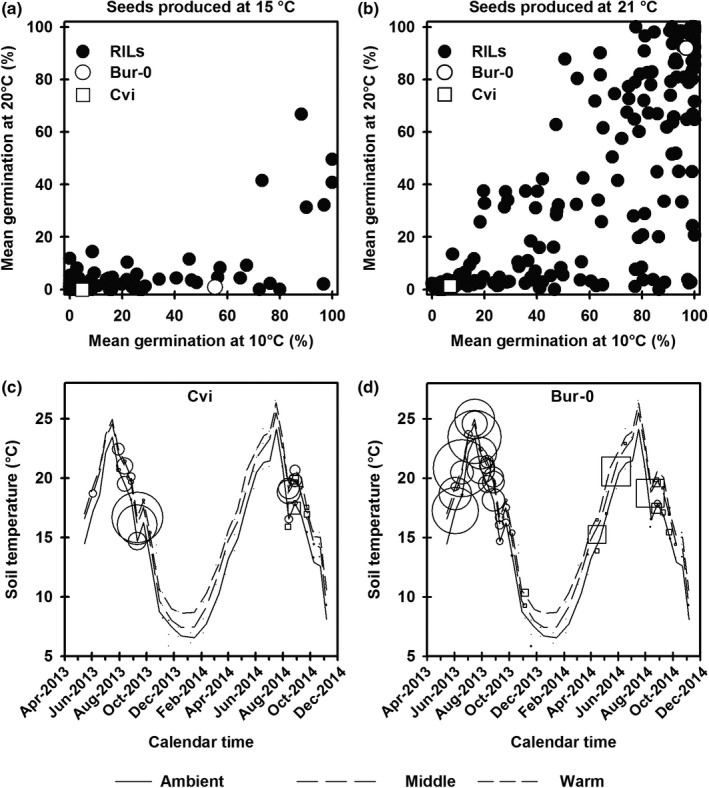
*Arabidopsis thaliana* seed germination and seedling emergence traits in a Burren (Bur‐0) × Cape Verde Islands (Cvi) recombinant inbred line (RIL) mapping population. Seed germination at 10 and 20°C in the parents and in 182 RILs produced at (a) 15 and (b) 21°C. (c, d) The annual pattern of temperature is shown for the ambient end (solid line), middle (dashed line) and warm end (dotted line) of the thermogradient tunnel. Circles and squares represent the timing of seedling emergence for the (c) Cvi and (d) Bur‐0 parents of the RIL population. Circle and square are emergence following spring and autumn dispersal, respectively; the larger the symbol, the greater the degree of seedling emergence.

**Figure 4:**
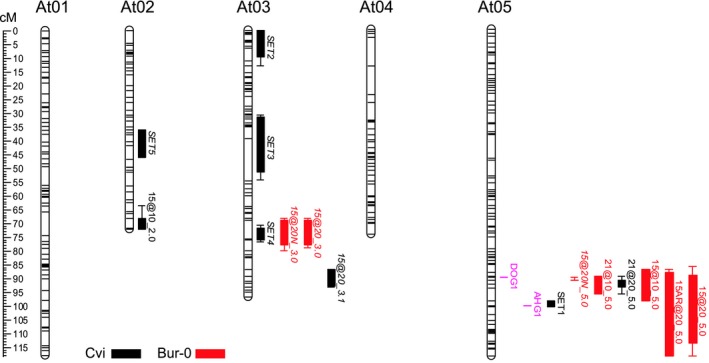
Quantitative trait loci (QTL) responsible for depth of dormancy in controlled environments, and the timing of *Arabidopsis thaliana* germination and seedling emergence during annual dormancy cycling in the field. QTL for seedling emergence timing (SET) and for germination of seeds produced at 15 and 21°C germinated at both 10 and 20°C were identified. Further QTL for germination following after‐ripening (AR) and in the presence of nitrate (N) were identified with seeds produced at 15°C. QTL name first shows the production temperature with AR when used, then germination temperature with N, when used, and chromosome number. For example, 15@20_3.0, but then 15@20_3.1 for a second QTL on the same chromosome. QTL shown in red have Bur‐0 as the genotype increasing germination; those in black have Cvi as the genotype increasing germination. Boxes indicate 1.5 LOD confidence intervals on the cM scale shown. DOG1 and AHG1 loci are shown.

To define more directly the genetic control of different seasonal patterns of gene expression and field emergence, we constructed a new RIL mapping population from a cross between the ecotypes Cvi and Bur‐0 shown above to have contrasting annual seasonal cycles (Fig. 1). This population was screened for germination and dormancy traits in controlled environments and seedling emergence timing traits in variable environments (Fig. 3). Trait data were then analysed and line means used for QTL analyses (Fig. 4; Table S2).

### QTL analysis of depth of dormancy in controlled constant environments

To identify QTL for depth of dormancy (potential to germinate at a given temperature) we screened seeds and plants of the Cvi × Bur‐0 F_8_ RIL population. To investigate the effect of production temperature we screened seeds produced at both 15 and 21°C and found those produced at 15°C to be significantly more dormant than those produced at 21°C (Fig. 3a,b). As Arabidopsis seeds are thermodormant, each of these productions were screened for germination at 10 and 20°C to determine the effect of germination temperature (Fig. 3a,b); both common and distinct QTL were identified (Fig. 4; Table S2). Bur‐0 seeds preferentially germinate at the higher temperature, whereas Cvi preferentially germinate at the lower temperature (Fig. 3a,b; Footitt *et al*., 2013). The RIL population expressed a full spectrum of germination phenotypes, including transgressive segregation extending beyond the parental means.

QTL with overlapping support intervals were located on chromosome 5 (At5) for all germination traits co‐located with *DOG1* (Bentsink *et al*., 2010). These co‐locating QTL on At5 dominated the potential to germinate (depth of dormancy) with LOD scores of, for example, 21.2 and 28.2 (43% and 53% of explained variance, respectively) at 10 and 20°C respectively for seeds produced at 21°C (Fig. 4; Table S2). At 20°C the genotype increasing germination (increasing genotype) was Cvi, whereas at 10°C, which facilitated a greater percentage germination, it was Bur‐0. In the deeper dormant seeds produced at 15°C and germinated in the presence of nitrate the LOD score was 44.4 (68% of explained variance; increasing genotype Bur‐0). In contrast, following after‐ripening (AR) of seeds produced at 15°C, the LOD score was 4.7 only (11% of explained variance; increasing genotype Bur‐0). Further minor QTL were identified. Seeds produced at 15°C with germination at 10°C revealed a QTL on At2 with a LOD score of 2.5 (5% of explained variance; increasing genotype Cvi), whereas germination at 20°C revealed a further two QTL on At3 (one co‐locating with *DOG6,* Bentsink *et al*., 2010) with LOD scores of 3.6 and 4.3 respectively (8% and 4% of explained variance and increasing genotype Bur‐0 and Cvi respectively).

### QTL analysis of seedling emergence timing in a seasonal environment

We sowed seed of 86 RILs (F_9_) chosen to represent the range of characteristics seen in the full population produced at 15°C at two times of year to represent seeds shed in spring and in autumn. These were sown at three positions within a thermogradient tunnel at low (ambient temperature), middle (*c. *ambient + 2°C), and warm (*c. *ambient + 4°C) temperatures (Fig. 3c,d) to cover the range of projected global warming at this location through to 2080 (Huang *et al*., 2018). The soil was disturbed every 2 wk to expose buried seeds to light to remove the final layer of dormancy when the ‘seeds became sensitive; nitrate was naturally present in the soil (3.12 ± 0.51 mg (kg DW^−1^)) and was therefore not a treatment. Time to seedling emergence (which includes germination) was recorded, and characteristic seedling emergence patterns for a summer and winter annual were exhibited by Bur‐0 and Cvi parent lines respectively (Fig. 3c,d). A full range of patterns between these two were exhibited by the 86 RILs selected to cover the full range of dormancy seen in the wider population.

To quantify seedling emergence patterns (e.g. Fig. 3c,d) for the identification of seedling emergence timing (*SET*) QTL, we calculated the relative proportion of seedling emergence that occurred when temperature was falling following both autumn and spring sowings. This measure, ‘emerge’, characterized the tendency to be a winter annual, and five *SET* QTL were identified. The most significant QTL was on At5 (LOD 17.3; *SET1*), accounting for 46% of the explained variance. This was close to, but confidence intervals did not include, *DOG1* (Fig. 4; Fig. S1; Table S2). When marker physical coordinates were plotted against coordinates in the genetic map of At5, a near linear order was present, suggesting a close relationship between physical and genetic distances (Fig. S1). These data confirm that the QTL *SET1* is independent of *DOG1*, and thus *DOG1* did not impact *SET*.

Further QTL were also identified. There were three separate QTL on At3, each collocating with different DOG QTLs identified by Bentsink *et al*. (2010): *SET2* (LOD 3.2; *DOG22*), *SET3* (LOD 3.5; *DOG21*), and *SET4* (LOD 12.6; *DOG6*). These accounted for 6%, 17% and 29% of the explained variance, respectively. A further QTL, *SET5*, was identified on At2 (LOD 3.8), and this collocated with *DOG20* (Bentsink *et al*., 2010). All of these QTL had Cvi as the increasing genotype. We looked for interactions between the *SET* QTL, identified as involved in the control of dormancy cycling. There was a large effect of *SET1* and *SET4*, with a smaller effect of *SET2* even after fitting the former two. There were no interactions, so the genetic effects were roughly additive on the logit scale.

## Discussion

### DOG1 determines depth of dormancy at maturity, but not the post‐shedding annual dormancy cycle that determines timing of seedling emergence

We identified QTL for depth of dormancy in a new RIL mapping population and confirmed previous results (Bentsink *et al*., 2006) that *DOG1* was the principle QTL in line with its protein determining depth of dormancy at the end of maturity. Consistent with this we identified the same QTL when seeds were produced both at 15 and 21°C, despite the far deeper dormancy across lines in the former. Different minor QTLs were also identified when the seeds produced were germinated at 10 or 20°C, indicating that the manifestation of dormancy at different temperatures is differently regulated in this thermodormant species.

However, the principle aim of this work was to gain greater understanding of the regulation of the annual dormancy cycle in seeds which determines the timing of the crucial phase transition to seedling establishment and growth. We studied genome‐wide expression patterns during annual dormancy cycling in two ecotypes (Bur‐0 and Cvi) with contrasting annual cycles. These patterns were summarized into nine clusters, with patterns linked to shallow and deep dormancy, environmental conditions and the potential for germination. As *DOG1* had previously been causally linked to seedling emergence timing (Huang *et al*., 2010; Postma & Agren, 2016; Finch‐Savage & Footitt, 2017), we looked specifically at genes previously identified as having *DOG1*‐dependent gene expression (Bentsink *et al*., 2010; Dekkers *et al*., 2013) in both Cvi and Bur‐0. Genes that were up‐regulated in the more dormant *NILDOG1* compared to *Ler* (Bentsink *et al*., 2010) were more highly expressed in Cvi (deep dormant) in the winter than in Bur‐0 (shallow dormant) (Fig. 2a). By contrast, those genes up‐regulated in *Ler* were more highly expressed in Bur‐0 than Cvi in the summer. Importantly this shows that the consequences of altered DOG1 activity are not limited to the period of high *DOG1* expression, and suggests that summer gene expression can be affected by the levels of gene expression in the winter. Furthermore, it shows that the pattern of *DOG1* expression is highly relevant to the annual gene expression linked to changing depth of dormancy. Nevertheless, although our QTL analysis clearly indicated a key role for *DOG1* in determining primary dormancy depth and in the regulation of maturation‐associated genes, it did not support a direct role for *DOG1* in generating altered seasonal patterns of gene expression between the two ecotypes. Crucially, this is independently supported by the analysis of global expression patterns where *DOG1* was confined to a temperature dependent cluster (cluster 2) rather than an ecotype dependent cluster. A new QTL, *SET1*, was identified as the principle regulator of seedling emergence timing. The close proximity of *SET1* and *DOG1* on chromosome 5 suggests the presence of a contiguous region forming a dormancy regulon. In support of this proposition, of the 363 genes in the interval including *DOG1* and *SET1*, 45 appear in ABA response or seed related GO categories.

We show here when plotting the physical coordinates that *SET1* is clearly distinguishable from *DOG1* as a distinct QTL in our analysis (Fig. S1); we therefore looked at the intervals defining the emergence QTL collocating with *DOG1* in previous work (Huang *et al*., 2010; Postma & Agren, 2016). Although it is difficult to compare directly since different mapping populations with associated genetic maps having different marker intervals were used, the presence of greater mean intervals around the QTL (> 8.6 cM) compared to *SET1* here at 2.3 cM suggests that in the previous work the confidence intervals of the *SET1* and *DOG1* QTL would overlap, and those analyses likely lacked the power to distinguish the two QTLs. Indeed, it may have been that *SET1* was not segregating in those studies; however, Montesinos *et al*. (2009) show that many ecotypes have indistinct annual cycles that morph into winter or summer annual behaviour depending on the environment. Here the construction of a mapping population from parents selected to have contrasting obligate winter and summer annual behaviour, compared to, for example, the two winter annuals used elsewhere (Postma & Agren, 2016), maximised phenotypic variation, with extremes in the observed phenotypes providing enhanced detection and resolution of QTL determining different seedling emergence patterns.

### Candidate genes underlying QTLs for dormancy cycling and seedling emergence timing (SET)

Current knowledge of gene function in the literature can be applied to justify selection of clear candidate genes underlying *SET* QTL and therefore the regulation of dormancy cycling. Although *SET1* does not collocate with *DOG1*, the other four *SET* QTL collocated with *DOG*s *20, 21*,* 22* and *6*. The latter is particularly interesting since it collocated with the second most significant QTL for both depth of dormancy and emergence timing (*SET4*). It was also collocated with a QTL for seedling emergence timing by Huang *et al*. (2010). The gene underlying *DOG1* has been cloned as At5g45830 (Bentsink *et al.*, 2006) and the likely candidate gene for *DOG6* has been identified as *ANAC060*, *At3g44290* (He, 2014). GO functions identified both as having DNA binding activity – the former sequence‐specific DNA binding, and the latter DNA‐binding transcription factor activity. The genes underlying *SET1*,* 2*,* 3*, and *5* QTLs are not known. The RNA‐Seq analysis described above (Figs 1 and 2), in addition to previous work, shows that neither DOG1 nor the concentration of ABA directly determine seedling emergence timing, but both are required (reviewed by Finch‐Savage & Footitt, 2017). Control appears to come from changing sensitivity to ABA linked to the amount of DOG1 present. Large amounts of DOG1 and high ABA concentrations in deep dormancy prevent germination, whereas reduced DOG1, ABA and sensitivity to ABA in shallow dormancy result in an increasing sensitivity to light that removes the final layer of dormancy to allow germination completion (Footitt *et al*., 2011). Thus, the characteristic profile of candidate genes controlling seedling emergence timing is that they should interact with DOG1 and influence ABA sensitivity, and because *DOG1* expression does not differ, the expression of the candidate is likely to differ in the Bur‐0 and Cvi ecotypes to enable the generation of their characteristic germination timings (seedling emergence).

In each of the regions defined by markers linked to *SET1* to *5* QTLs, there are 92, 57, 26, 82 and 64 genes respectively (Table S3). Analysis of GO for these genes with roles in ABA responses and seed related functions indicated that each *SET* QTL contains genes with functions assigned to these categories (Table S4). Individually *SET1* (45%), *SET4* (*29%*) and *SET3* (17%) accounted for much of the explained variance in seedling emergence timing, and so we concentrated on these three QTLs. *SET3* and *4* have clear candidate genes. In *SET3* there was only one gene with an appropriate GO categorisation (*PROTEIN PHOSPHATASE 2A SUBUNIT A2* (*PP2AA/PDF1*)), which acts upstream of *DOG1*. *PDF1* encodes one of the three scaffolding subunits of the PP2A family (Zhou *et al*., 2004) and has been previously shown to have a negative role in seed dormancy (Nee *et al*., 2017). *SET4* collocates with *DOG6*, with the likely candidate gene *ANAC060*; At3G44290 (He, 2014). ANAC060 does not appear in seed related GO categories (Table S4); however, it is in the GO category ‘Cellular response to glucose stimulus’. Both of these genes have the characteristic profile for candidate genes controlling seedling emergence timing.

**Figure 5:**
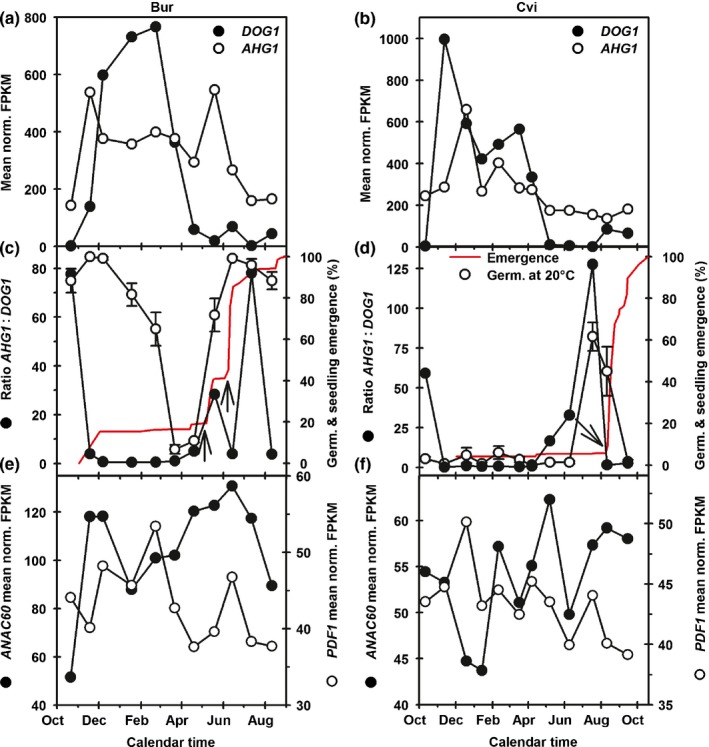
Expression of candidate genes in *Arabidopsis thaliana*. Annual expression profiles of candidate genes in the Burren (Bur‐0) (a, c, e) and Cape Verde Islands (Cvi) (b, d, f) ecotypes. (a) *DOG1* and *AHG1*. (c, d) The profile of the ratio of *AHG1* : *DOG1* expression over the annual cycle mirrors the increasing sensitivity to light allowing germination completion in both ecotypes. An increased ratio is followed by seedling emergence recorded on plots that were regularly disturbed to expose seeds to light. Such exposure completes dormancy loss if seeds have become sensitive to light in the annual cycle. Arrows indicate rainfall coincident with the start of seedling emergence. (e, f) Annual expression profiles of *ANAC060* and *PDF1*. Error bars indicate ± SE; absence indicates SE is smaller than the symbol. Germ., germination; norm. FPKB, normalized fragments per kilobase.

*ANAC060* (*DOG6; SET4*) exhibited an annual expression pattern that differed from that of *DOG1* (Fig. 5). This is consistent with their different functions, since DOG1 increases sensitivity to ABA and enhances dormancy (Finch‐Savage & Footitt, 2017), whereas ANAC060 reduces sensitivity to ABA (Li *et al*., 2014). *ANAC060* expression is induced by the sugar‐ABA signalling cascade and normally results in sugar sensitivity. However, in Col‐0 a 20 base pair insertion before the final exon that encodes an in‐frame stop codon results in a truncated protein that is retained in the nucleus, where it reduces glucose‐induced ABA accumulation and *ABI4* expression, reducing sensitivity to ABA (Li *et al*., 2014). This 20 base pair insertion is present in ANAC060 in both Cvi and Bur‐0 (data from 1001 genomes project; http://1001genomes.org). Both act at the level of DNA binding; *DOG1* expression was correlated (negatively) to the annual temperature cycle, but *ANAC060* was not. Potentially this behaviour provides a sensitive response to the environment that can differ between ecotypes. Recently, basic LEUCINE ZIPPER TRANSCRIPTION FACTOR67 (bZIP67) was shown to transactivate *DOG1* during maturation to help establish primary dormancy (Bryant *et al*., 2019). Interestingly this gene maps adjacent to *DOG6* and within *SET4*; however, consistent with its role in the induction of *DOG1*, its gene expression pattern peaks before or coincident with increased *DOG1* (Fig. S2) in both ecotypes and thus does not fulfil the criteria outlined for a candidate SET gene.

In the most significant QTL, *SET1*, we found 10 genes with an appropriate GO categorisation (Table S4). Three of these genes (At5G51990, At5G52050, At5G52200) were not represented in the RNA‐Seq data and were therefore not expressed during dormancy cycling. Four further genes (At5G51340, At5G51430, At5G52300, At5G52310) were significantly (*P* < 0.05) correlated with *DOG1* and temperature and therefore do not fit the criteria above for candidate genes whose expression patterns should differ between Cvi and Bur‐0. Of the remaining three genes (At5G51110, At5G51300, At5G51760), only the latter *AHG1* (Nishimura *et al*., 2007) interacts with DOG1 (Nee *et al*., 2017; Nishimura *et al*., 2018) and therefore fits all the criteria to make it a clear candidate gene. AHG1 is known to interact with DOG1, alter ABA sensitivity and have a seed dormancy phenotype, and it encodes a PP2C (protein phosphatase of the 2C family) with essential roles in the release of seed dormancy (Nee *et al*., 2017; Nishimura *et al*., 2018). AHG1 also interacts with DELAY OF GERMINATION‐LIKE 3 (DOGL3), with seeds over expressing *DOGL3* having delayed germination (Nishimura *et al*., 2018). During the annual dormancy cycle, the transcript profile of *DOGL3* was similar in magnitude and pattern to *DOG1*, with *DOGL3* levels somewhat higher than *DOG1* when dormancy is lowest (coincident with high germination potential; Fig. S3). Like *DOG1*,* DOGL3* is not within a SET QTL.

A second PP2C, AHG3, was also found to interact with DOG1 by Nee *et al*. (2017). Both were epistatic to DOG1, altered sensitivity to ABA and were considered by Nee *et al*. (2017) to be the likely point at which ABA and DOG1 pathways converge in the regulation of dormancy. Thus, unlike *PDF1* (identified as a candidate above in SET3), which acts upstream of DOG1, AHG1 and AHG3 act downstream of DOG1. Thus, enhanced levels of DOG1 and absence of AHG1 and 3 in the double mutant *ahg1 ahg3* both lead to enhanced dormancy (Nee *et al*., 2017). AHG1 and AHG3 were shown to be redundant, with AHG1 being the dominant allele (Nee *et al*., 2017), and this is consistent with AHG3 not being present in any of the *SET* QTLs. Nishimura *et al*. (2018) show that AHG1 does not bind to the ABA receptor protein PYR1 to alter the ABA response, but AHG3 and some other PP2Cs do. Thus, Nishimura *et al*. (2018) proposed a model whereby AHG3 and other PP2Cs down‐regulate ABA signalling via a pathway independent of DOG1/AHG1. This provides the intriguing possibility that DOG1‐regulated ABA sensitivity via AHG1 could operate in the deep dormancy phase of cycling, while AHG3 binding to the PYR1 receptor could regulate dormancy in the shallow phase where *PYR1* expression is highest (Footitt *et al*., 2011, 2013). The *ANAC060*,* AHG1* and *PDF1* expression patterns differed strongly between the two ecotypes and differed from that of *DOG1* (Fig. 5a,b,e,f).

DOG1 acts upstream to suppress the action of AHG1, and crucially *DOG1* expression is linked to the same pattern of seasonal temperature in both ecotypes studied; thus, as *DOG1* expression decreases towards zero with likely reduced protein activity, it would be the presence of AHG1 that would reduce ABA sensitivity to initiate dormancy loss leading to germination and seedling emergence. We therefore considered the relative amounts of expression of the two genes. An increase in the *AHG1* : *DOG1* ratio coincided with increasing germination potential in the population. Combined with the known modification of the DOG1 protein as dormancy declines (Nakabayashi *et al*., 2012), this indicates that their dynamic relationship at the protein level is changing. Although regulation occurs at the protein level, the *AHG1* : *DOG1* ratio increased in Bur‐0 coincident with germination of recovered seeds in the laboratory and crucially with the two flushes of seedling emergence seen in the field (Fig. 5c,d). Furthermore, in Cvi the ratio increased coincident with maximum germination of recovered seeds and before seedling emergence in the field. Seedling emergence in the field requires adequate soil moisture as well as exposure of the seed to light. In both ecotypes seedling emergence therefore coincided with rainfall (Fig. 5c,d) following an increase in the *AHG1* : *DOG1* ratio.

In support of this possible mechanism of DOG1 repression of AHG1, we also looked at the ratio of *AHG1* : *DOG1* expression in the data of Cadman *et al*. (2006), who measured gene expression in a range of fixed dormant states of different depths that form the building blocks of dormancy cycling. The ratio was very low in deeply dormant states and increased progressively in states with shallower dormancy (Fig. S4), confirming what we observe here. Furthermore, and consistent with this, *AHG1* was expressed in the newly imbibed seeds, but not as seeds progressed to germination completion (Fig. S4). During dormancy cycling *PDF1* expression level was similar to *ANAC060* (Fig. 5e,f), and interestingly the ratio of these two genes to *DOG1*, although of lower magnitude, had the same pattern as *AHG1* : *DOG1*, with peaks coinciding with germination and seedling emergence (Fig. S5).

### A model for the regulation of dormancy cycling via negative responses to ABA

**Figure 6:**
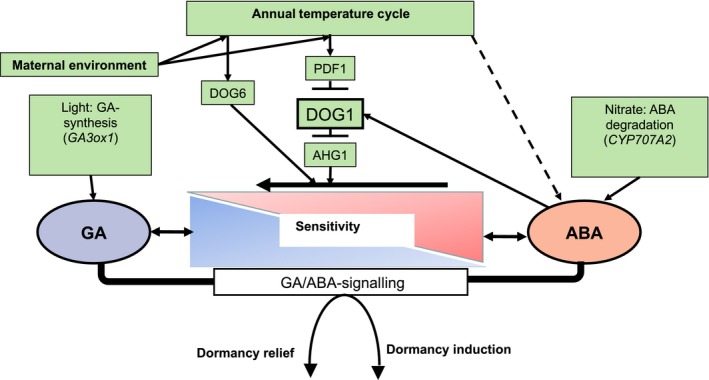
Schematic model for the regulation of dormancy cycling in *Arabidopsis thaliana*. Maternal environment affects DOG1 and DOG6 (ANAC060) to determine initial depth of dormancy. Their influence differs with accession; for example, the Cape Verde Islands (Cvi) ecotype is known to have a very strong dominating DOG1 allele (Bentsink *et al*., 2006). *ANAC060 and DOG1* (*DOG6*) expression patterns alter during the annual cycle, and the DOG1 response is thought to be anchored to that of temperature, acting as a means of accumulating thermal time (Footitt *et al*., 2015; Finch‐Savage & Footitt, 2017). PDF1 acts upstream of DOG1 to reduce depth of dormancy, and may therefore facilitate the DOG1 environmental response (Nee *et al*., 2017). In winter DOG1 expression is high, AHG1 action is suppressed by DOG1, and seeds are not sensitive to spatial signals. During spring DOG1 expression decreases to reduce suppression of AHG1, with a concurrent reduction in sensitivity to abscisic acid (ABA). Presence of AHG1 therefore determines the timing of subsequent germination and seedling emergence. In response there is increased sensitivity to spatial signals (light and nitrate) that further alter the hormone balance and remove the final layer of dormancy in favour of germination completion. In addition, ANAC060 (*DOG6*) appears to act at the same level (DNA binding) as DOG1, but where DOG1 increases sensitivity to ABA by inhibiting AHG1, ANAC060 reduces sensitivity (Li *et al*., 2014).

Both ANAC060 and AHG1 reduce sensitivity to ABA, but activity of AHG1 is repressed by DOG1 (Nee *et al*., 2017); this is not true of ANAC060. Thus, ANAC060 would tend to oppose the DOG1 influence on the hormone balance, and the relative importance of the two will differ between ecotypes. Indeed, all three candidate genes (AHG1, ANAC060 and PDF1) considered here negatively affect ABA sensitivity, suggesting that dormancy cycling is regulated via a negative response to ABA. This is in direct contrast to initial depth of dormancy, which is dominated by a positive response to ABA via DOG1. Based on the results presented here, as well as those of Nee *et al*. (2017) and Nishimura *et al*. (2018), we propose a model (Fig. 6) of the regulation of dormancy cycling as an extension to that of Finch‐Savage & Footitt (2017), centred on the hormone balance mechanism. We have shown that all of the proposed components of the model are present in the contrasting ecotypes compared. However, the responses of these components during the annual cycle differ in a way that is consistent with the ecotypes’ characteristic patterns of dormancy cycling, and their timings of germination completion and subsequent seedling emergence.

## Author contributions

WEF‐S and SF planned and designed research. SF, AJH, SP and WEF‐S performed experiments. SF, SP, PGW and JRL analysed data. WEF‐S, SF and SP wrote the manuscript, with additions by PGW and JRL.

## Supporting information

Please note: Wiley Blackwell are not responsible for the content or functionality of any Supporting Information supplied by the authors. Any queries (other than missing material) should be directed to the *New Phytologist* Central Office.

**Fig. S1** Marker physical coordinates plotted against coordinates in the genetic map of At5.
**Fig. S2** Expression patterns of *DOG1* and *bZIP67* in *Arabidopsis thaliana* ecotypes Burren (Bur‐0) and Cape Verde Islands (Cvi).
**Fig. S3** Expression patterns of *DOG1* and *DOGL3* in in *Arabidopsis thaliana* ecotypes Burren (Bur‐0) and Cape Verde Islands (Cvi).
**Fig. S4** The ratio of *AHG1* : *DOG1* in the data of Cadman *et al*. (2006).
**Fig. S5** The ratio of *PDF1* and *ANAC060* to *DOG1* over an annual cycle.
**Methods S1** Additional materials and methods.

**Table S1** Details of genes shown in nine clusters identified by RNA‐Seq analysis (Fig. 1).

**Table S2** Quantitative trait loci identified for traits describing measures of germination and the speed of germination in the Cape Verde Islands (Cvi) × Burren (Bur‐0) F_8_ recombinant inbred line mapping population.

**Table S3** Details of genes present in *SET* quantitative trait loci (Fig. 4).

**Table S4** GO analysis of genes in *SET* quantitative trait loci.
