# Supplementary material for: Trait analysis reveals DOG1 determines initial depth of seed dormancy, but not changes during dormancy cycling that result in seedling emergence timing
Source: New Phytol. 2019 Sep 18;225(5):2035–47. doi: 10.1111/nph.16081 (PMC7027856; doi:10.1111/nph.16081)
Supplement: Supplementary file 1 — Fig. S1 Marker physical coordinates plotted against coordinates in the genetic map of At5. Fig. S2 Expression patterns of DOG1 and bZIP67 in Arabidopsis thaliana ecotypes Burren (Bur‐0) and Cape Verde Islands (Cvi). Fig. S3 Expression patterns of DOG1 and DOGL3 in in Arabidopsis thaliana ecotypes Burren (Bur‐0) and Cape Verde Islands (Cvi). Fig. S4 The ratio of AHG1 : DOG1 in the data of Cadman et al. (2006). Fig. S5 The ratio of PDF1 and ANAC060 to DOG1 over an annual cycle. Methods S1 Additional materials and methods. [file NPH-225-2035-s001.pdf]

New Phytologist Supporting Information Figs S1-S5 and Methods S1

Article Title: Trait analysis reveals DOG1 determines initial depth of dormancy, but not changes during dormancy cycling that result in seedling emergence timing.

Steven Footitt, Peter G. Walley, James R. Lynn, Angela J. Hambidge, Steven Penfield and William E Finch-Savage

Article acceptance date: 20 July 2019

Figure S1. Relationship between physical distance (Mbp) and genetic diatance (cM) on chromosome At05.

Marker physical coordinates were plotted against their coordinates in the genetic map, a near linear order was present, suggesting a close relationship between physical and genetic distances (Figure x insert). The chromosomal region containing *DOG1* and *SET1* (shaded region of graph on insert) was selected, and indeed the QTL *SET1* is independent of *DOG1* and contains the phosphatase *AGH1*.

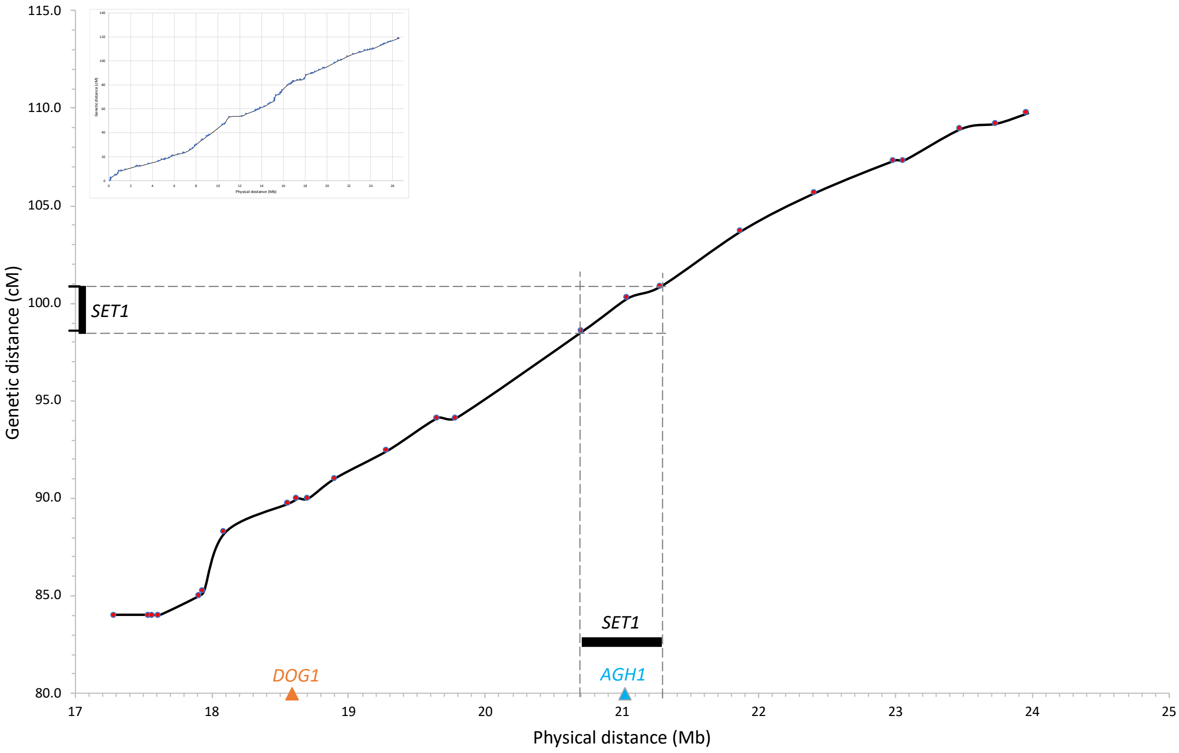

Relationship between physical distance (Mbp) and genetic distance (cM) on chromosome At05. The QTL SET1 is indicated by a black bar delimited by hashed lines. The physical coordinates for *DOG1* (orange triangle) and *AHG1* (blue triangle) are highlighted.

Figure S2. Expression of *DOG1* and its trans-activator *bZIP67* during dormancy cycling in field soils. Relative gene expression levels of *DOG1* and *bZIP67* in the *Arabidopsis* ecotypes (a) Bur and (b) Cvi during dormancy cycling in field soils. The correlation coefficients for *DOG1* vs *bZIP67* are -0.026 (not significant) (Bur) and 0.59 ($p<0.05$) (Cvi). The large increases in *bZIP67* seen in autumn are immediately prior to increases in *DOG1* seen in both Bur and Cvi in October and November respectively the periods not covered by the RNAseq (see Fig. 8 and Footitt S, Huang Z, Clay H, Mead A, Finch-Savage WE. 2013. Temperature, light and nitrate sensing coordinate *Arabidopsis* seed dormancy cycling resulting in winter and summer annual phenotypes. *The Plant Journal* 74: 1003-1115).

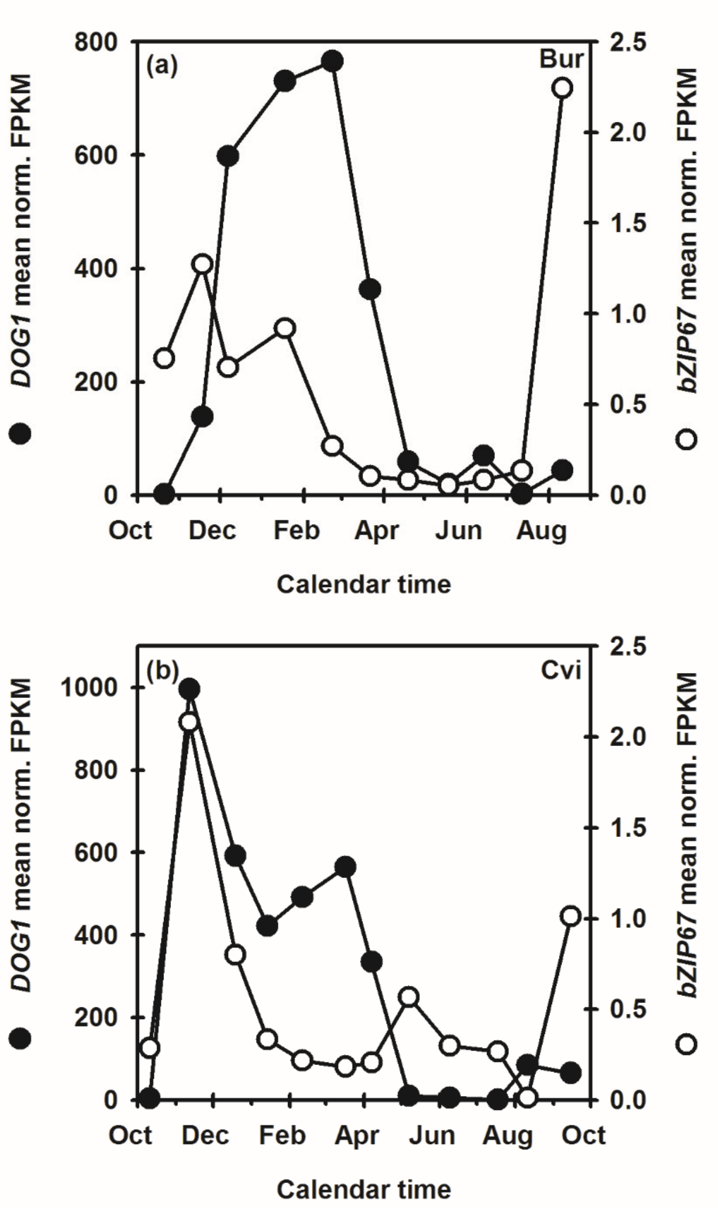

Figure S3. Expression of *DOG1* and *DOGL3* during dormancy cycling in field soils. Relative gene expression levels of *DOG1* and *DOGL3* in the *Arabidopsis* ecotypes (a) Bur and (b) Cvi during dormancy cycling in field soils. The correlation coefficients for *DOG1* vs *DOGL3* are -0.793 ($p<0.01$) (Bur) and 0.54 (not significant) (Cvi). Both DOG1 and DOGL3 proteins interact physically with AHG1 with DOGL3 potentially providing an additional level of control on AHG1 activity (Nishimura N, Tsuchiya W, Moresco JJ, Hayashi Y, Satoh K, Kaiwa N, Irisa T, Kinoshita T, Schroeder JI, Yates III JR, et al. 2018. Control of seed dormancy and germination by DOG1-AHG1 PP2C phosphatase complex via binding to heme. *Nature communications* 9: 2132-2132).

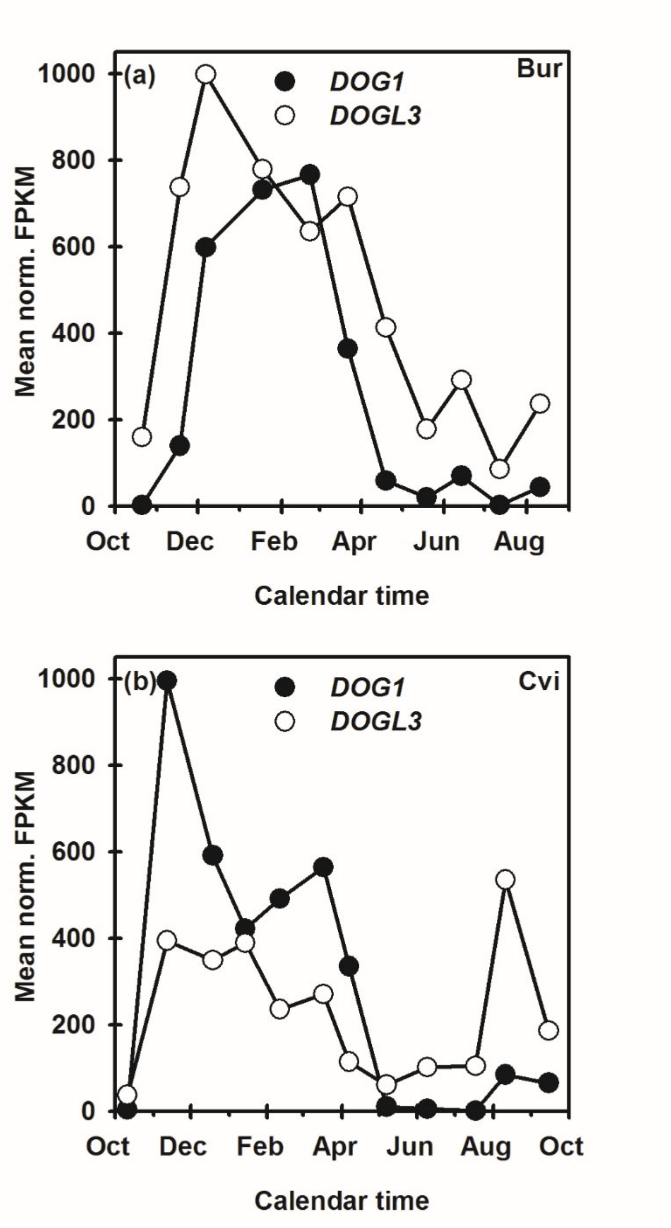

Figure S4. Gene expression of *AHG1,* AHG3 and *DOG1* in dormant *Arabidopsis* seeds and *AHG1* during germination. (**a**) Relative gene expression of *AHG1,* AHG3 and *DOG1* in seeds with different levels of dormancy. (**b**) The ratio of *AHG1* + *AHG3/DOG1* in seeds with different levels of dormancy. (**c**) Microarray data of *AHG1* gene expression in the radicle and micropylar endosperm tissues of germinating *Arabidopsis thaliana* seeds (Dekkers BJW, Pearce S, van Bolderen-Veldkamp RP, Marshall A, Widera P, Gilbert J, Drost HG, Bassel GW, Muller K, King JR et al. 2013. Transcriptional dynamics of two seed compartments with opposing roles in *Arabidopsis* seed germination. *Plant Physiology* 163: 205-215). Data in (**a**) and (**b**) are from the transcriptome analysis of Cadman et al. (Cadman CSC, Toorop PE, Hilhorst HWM, Finch-Savage WE. 2006. Gene expression profiles of *Arabidopsis* Cvi seeds during dormancy cycling indicate a common underlying dormancy control mechanism. *The Plant Journal* 46: 805-822) and data are available from Arabidopsis efp browser (http://bar.utoronto.ca/efp/cgi-bin/efpWeb.cgi). In (**c**) data available from the Nottingham eFP browser (http://ssbvseed01.nottingham.ac.uk/efp_browser/efpWeb.cgi). Descriptors for dormancy states in (**a**) and (**b**): PDD, Primary dormant: seeds dry (will not germinate when imbibed in Light or dark). DDL, Primary dormant seeds, dry after-ripened: seeds dry (will germinate when imbibed in the light). PD24h, PD48h, and PD30d, Primary dormant seeds imbibed in the dark for 24 or 48 hours, or 30 days (will not complete germination). SD1, Secondary dormant: DL seeds imbibed in the dark for a further 24 days (sensitive to nitrate). SD2, Secondary dormant: SD1 seeds imbibed at 3°C in the dark for 20 days (insensitive to nitrate). PDC, Primary dormant seeds after-ripened for 117 days and then imbibed for 4 days at 3°C (will complete germination if exposed to light). PDL, Primary dormant seeds after-ripened for 91 days and then imbibed for 24 h in the light (will not complete germination). DL, dry after-ripened seeds imbibed for 24 h (will germinate if placed in the light). PDN, Primary dormant seeds after-ripened for 91 days and then imbibed for 24 h on a 10 mM KNO3 solution (will complete germination if exposed to light). PDLN, Primary dormant seeds after-ripened for 91 days and then imbibed in white light for 24 h on a solution 10 mM KNO3 (will complete germination). LIG, Dry after-ripened seeds imbibed for 20 h in the dark and then 4 h in red light (will complete germination).

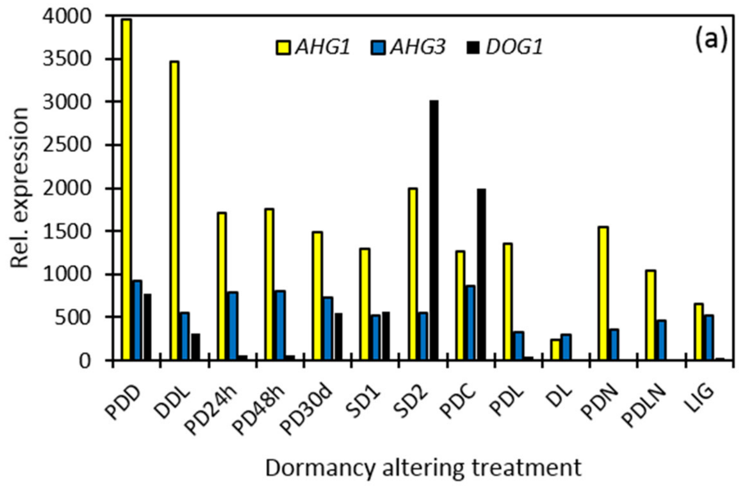

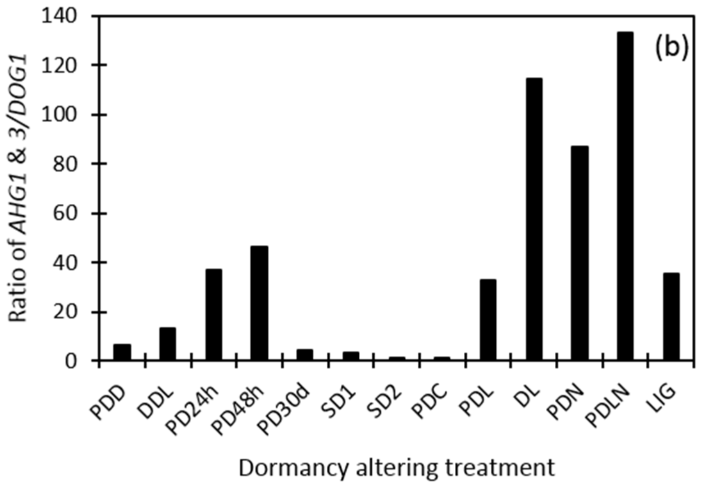
(b)
Ratio of AHG1 & 3/DOG1
140
120
100
80
60
40
20
0
PDD
DDL
PD24h
PD48h
PD30d
SD1
SD2
PDC
PDL
DL
PDN
PDLN
LIG
Dormancy altering treatment

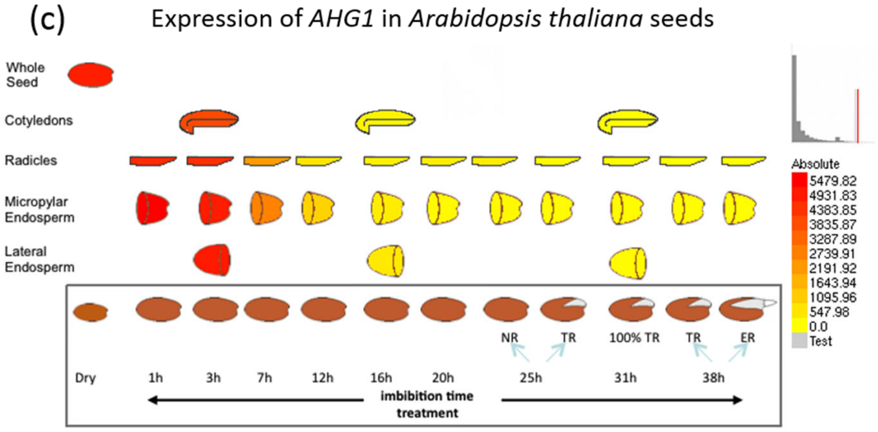
(c)
Expression of AHG1 in Arabidopsis thaliana seeds
Whole Seed
Cotyledons
Radicles
Micropylar Endosperm
Lateral Endosperm
Absolute
5479.82
4931.83
4383.85
3835.87
3287.89
2739.91
2191.92
1643.94
1095.96
547.98
0.0
Test
NR
TR
100% TR
TR
ER
Dry
1h
3h
7h
12h
16h
20h
25h
31h
38h
imbibition time
treatment

Figure S5. The ratio of *ANAC60/DOG1* and *PDF1/DOG1* during dormancy cycling in field soils. Ratio of gene expression levels of the candidate Seedling Emergence Timing (SET) genes, *ANAC60* and *PDF1,* versus *DOG1* are shown in the *Arabidopsis* ecotypes (a) Bur and (b) Cvi during dormancy cycling in field soils. Seedling emergence in the field and germination of recovered seeds at 20 °C/light are shown to indicate the changing level of dormancy and increased sensitivity to environmental signals such as light. Error bars indicate SE, absence indicates SE is smaller than the symbol

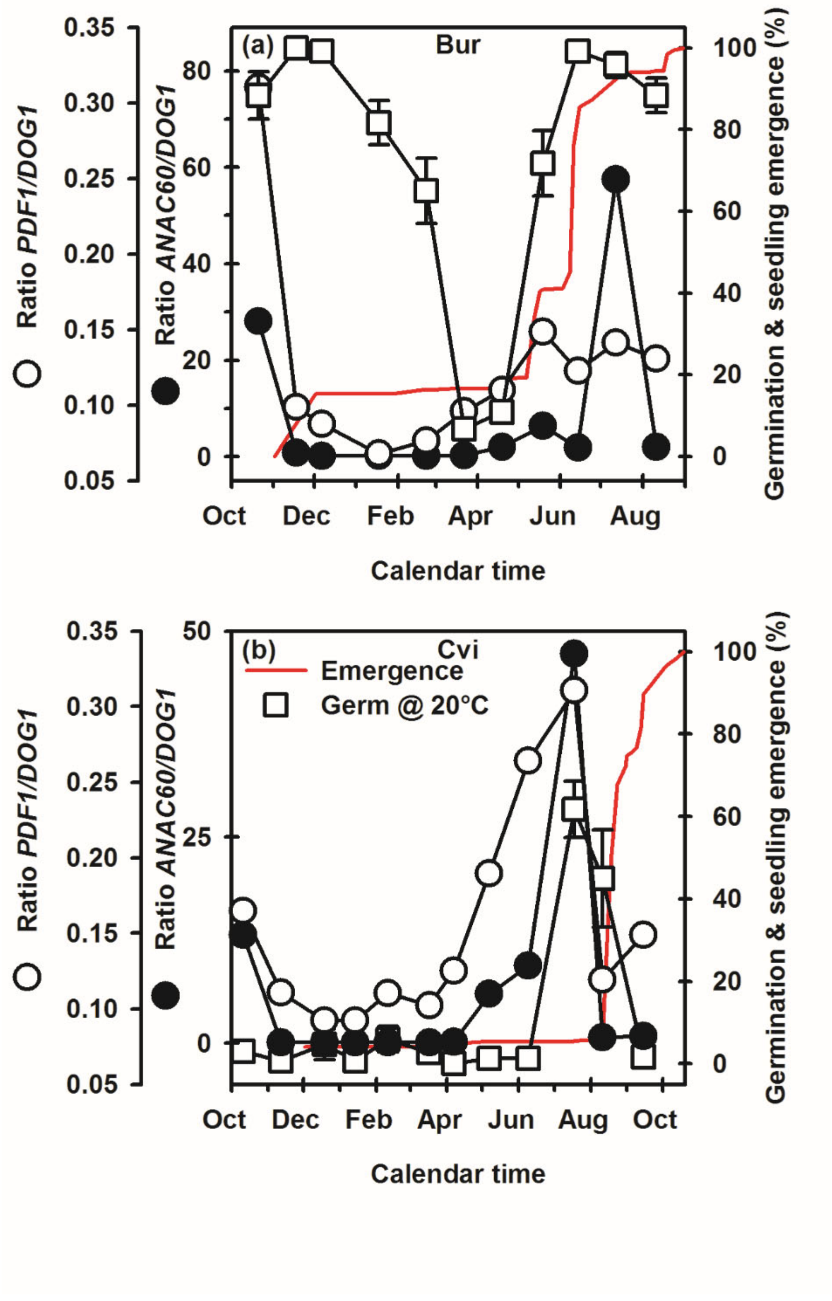

Methods S1

Seed Production

Seeds of the *Arabidopsis thaliana* ecotypes Cape Verdi Islands (Cvi; N8580) and Burren (Bur-0; CS6643) were produced (Cvi, February–May 2007; Bur-0, May-July 2009) in a temperature-controlled glasshouse (16–18 °C/ 16-h day: 10–15 °C/8 h night) with supplementary lighting (400 W high-pressure sodium lamps) (Osram) when light intensity fell below 300 W m2 during the 16-h day. Nondormant seeds of each ecotype were sown into compost (Levingtons F2/sand/vermiculite at a ratio of 6/1/1) in P24 cellular trays (24 cells, each 5 × 5 × 5 cm) held in capillary matting-lined seed trays. A single seedling in each cell was grown to maturity and mature seeds were harvested by hand threshing, then equilibrated at 15% relative humidity/15 °C for 7 d to produce an equilibrium moisture content of 5–7% on a dry-weight basis. Seeds were stored at −80 °C in sealed tubes.

Dormancy cycling in field soils to produce seed samples for RNAseq

Seeds for burial in 2007 (Cvi) and 2009 (Bur) were dispersed in Ballotini balls in nylon mesh bags (Potters Ballotini Ltd, Barnsley, UK) and buried in field plots at a depth of 5 cm in a randomised plot design as describe elsewhere (Footitt and Finch-Savage, 2011; Footitt *et al.* 2011). SM200 soil moisture sensors (Delta-T Devices) and Thermistore temperature probes (Betatherm, Galway, Ireland) linked to a data logger (Delta-T Devices Ltd, Cambridge, UK) recorded soil moisture and temperature at seed depth in dummy bags. Seeds for molecular analysis were recovered from the soil seed bank and flash frozen in liquid nitrogen in the dark using previously published procedures (Footitt and Finch-Savage 2011; Footitt *et al.* 2011). Seed dormancy of recovered seeds was determined as described previously (Footitt et al 2011; 2013).

Cvi X Bur Mapping population

The Cvi (maternal line) was crossed with Bur-0 (paternal line). The F1 seeds resulting from this cross were dry after-ripened at room temperature for two months then individual seeds sown into compost (Levingtons F2/sand/vermiculite at a ratio of 6/1/1) in P24 cellular trays (24 cells, each 5 × 5 × 5 cm) held in capillary matting-lined seed trays. The resulting plants were grown in temperature-controlled glass house (16–18 °C/ 16-h day: 10–15 °C/8 h night) with supplementary lighting (400 W

high-pressure sodium lamps) (Osram, Newton-le-Willows, UK) when light intensity fell below 300 W m2 during the 16-h day. On bolting each plant was isolated using an Aracon (Arasystem, Gent, Belgium) to prevent cross pollination and to facilitate seed collection, processing and storage as above. Seeds (F2) of one plant were selected and dry after ripened for 2 months at room temperature to reduce primary dormancy then used for single seed descent to the F8 generation under the above conditions. From the F2 generation onwards seeds were surface sterilized with chlorine gas as follows. Seeds of each line were held in Eppendorf tubes and kept in place with a detached lid containing a ventilation hole then placed in a desiccator containing a beaker with 100 ml household bleach to which 3 ml concentrated HCl was added. The desiccator was immediately sealed and left for 3 hours before venting in a fume cupboard. Eppendorf tubes containing seeds was sealed and stored at −80 °C until required.

F2 generation. Seeds (F2) were plated out in a laminar flow cabinet on to strips of sterile nylon mesh (mesh size 125 μm; Pastock, Birkenhead, UK) held in Petri plates containing 0.7% agarose and ½ strength MS salts at pH 5.8. Seed coats were then nicked with a Syringe needle to break dormancy. Plates were sealed with micropore tape and incubated at 15 °C under constant light. Following germination, seedlings were transplanted at the first true leaf stage to compost in trays as above in the glasshouse as above. Each seedling (line) was given a unique number. Trays were covered with propagator lids for seven days (vents were opened after 3 days) to aid seedling establishment. Following seed collection, 632 F3 lines were selected for the next round based on the distribution of bolting times (30 to 89 days).

F3 to F8 generation. Seeds were germinated as above (see F2 generation) with the following selection criteria to avoid biasing the population for low dormancy. Six seeds of each line were placed on nylon mesh in a Petri plate (50 mm dia.) and dormancy broken as above and incubated at 15 °C in the light. Any seeds not germinating were nicked again. Once all seeds had germinated, seedling 1, 3 and 5 were transplanted to compost as above (any seedling that died before transplantation at the true leaf stage was replaced with the adjacent seedling). The bolting date was recorded for each plant and the plant isolated with an Aracon; inflorescences were trimmed to stop them exiting the Aracon to prevent cross pollination. Plant 3 was used to obtain seeds of the next generation. If this plant died or was sterile, plant 1 was used, if this one failed then plant 5 was used (otherwise

they were discarded). This procedure was adapted from Alonso-Blanco et al. (1998). At the end of flowering each plant was allowed to undergo maturation drying before seed harvesting and storing as above. At each generation a decreasing number of lines were lost due to infertility.

F8 to F9 generation. Only F7 lines with F8 seed yields of 50 mg or greater were selected for the next generation. These were then ranked on bolting time and every second line eliminated then every third line eliminated to give a final recombinant inbred line population of 184 lines plus the parents. In this final population seed yield and bolting time were normally distributed. Eight F8 seeds were germinated using the above protocol, with seedling 1, 3, 5 and 7 selected for transplanting as above. Following transplanting, plants were grown to maturity in growth cabinets at two temperatures (15 °C and 21 °C under a 16 h light/8h dark cycle at a light intensity of 100 µmoles m^{-2} sec^{-1} and 80% RH (RH was gradually reduced to 45%)) to produce populations with different levels of dormancy. Two replicate plants were grown at each temperature. At bolting a rosette leaf was removed from each plant at 21 °C, frozen in liquid nitrogen and stored at -80 °C for genotyping. Total DNA was isolated using DNeasy plant maxi-kits (Quiagen Inc., Manchester, UK) following the manufacturers guidelines, and diluted to 100 ng µl-1 using TE (pH 8.0) and stored at -20 °C. The F_8 RIL population was genotyped using 400 KASP™ single nucleotide polymorphisms (SNPs) (LGC Genomics, Hoddesdon, UK), evenly spaced along the genome, and located in the open reading frame of a gene. Markers were discarded that did not segregate, were identical to an adjacent marker, were missing, heterozygous for one parent, or lay between a pair of identical markers while differing slightly from them. Then a linkage map of 347 SNPs was constructed using R/qtl (Broman et al. 2003).

When 2/3 of the siliques on a plant had turned yellow it was transferred to a separate tray and watering was stopped to allow plants to dry for 7 days. At harvest each plant was placed in a paper bag and dried in a drying room for 7 days at 15C/15% RH. Plants were then threshed and seeds cleaned, sealed in tubes and stored at -80°C.

Screening for seed dormancy in the F_9 generation

Following harvest and drying, approximately 400 seeds of each RIL and the parental lines were placed in 1.2Ml Eppendorf tubes and dry sterilised with chlorine gas (see

supplemental materials and methods) then stored at -80°C until required. Seeds were germinated in 12 X 8 cm boxes (Stewart Plastics Ltd, Banbury, UK) containing two pieces of Whatman 3MM chromatography paper (Camlab, Cambridge, UK) with one piece of blue blotter paper (Anchor Paper Co., St. Paul, MN, USA) on top and 16 mL water. The blue paper was marked into four sectors using a pencil. Each replicate RIL and parental line from the two production temperatures were tested separately. RILS and parental lines were randomly assigned to a sector on the blue germination paper. Into each sector was placed approximately 40 seeds by hand scattering. Then using forceps seeds were gently spread out so that none were touching. All boxes were numbered to identify the lines in each box. Boxes were sealed in freezer bags and incubated at 10 and 20°C in the light to evaluate the level of low and high temperature thermo-dormancy. In addition, seeds produced at 15°C were (a) incubated at 20°C in the presence of 10 mM KNO3 to evaluate nitrate sensitivity; and (b) were dry after-ripened at 20°C in the dark for 30 days then returned -80°C before testing germination at 20°C. This by comparison with germination at 20°C prior to after-ripening indicated the depth of dormancy in each line. Each test was repeated to give three independent replicates.

Germination recording and analysis used the GERMINATOR system as described in Joosen et al., (2010). Briefly, boxes containing seeds were placed on a copy stand illuminated on two sides by 30 mm white fluorescent tubes. Light was diffused through sheets of opaque white plastic that enclosed three sides of the copy stand, the open side was covered with a black velvet blackout curtain. Box position was fixed by a wooden template and the first images recorded prior to the onset of germination then at daily or twice daily intervals. A digital camera (Nikon D90 digital SLR with 60mm f2.8 G AF-s Ed Micro lens) linked to a computer and controlled by Nikon camera control pro software Version 2.0 was used to record images. Camera was set to manual control and settings were shutter speed 1/3 sec, aperture F14, and ISO 400. Image files were named using the convention described by Joosen et al (2010). Germination data was extracted from images as described in the GERMINATOR manual and data analysis used the excel GERMINATOR spread sheet (http://www.pph.wur.nl/UK/seedlab/resources/germinator/).

Final percentage germination data were analysed in Genstat (VSN International, 2013) using REML (VSN International, Hemel Hempstead, UK), following an empirical logit transformation, in which the data were adjusted by 0.5%

to move germination rates away from 0 and 100%. Replicate tests, box within tests, and a residual were taken as random factors. Means from this analysis were subjected to QTL analysis.

Seedling emergence of F_9 seeds under global warming scenarios in a thermogradient tunnel

Thermogradient tunnel: The polyethylene tunnel (32 m long × 9 m wide) structure enabled seedling emergence to be monitored under natural day lengths with a high percentage (76%) of natural irradiance levels. The ambient air temperature was constantly monitored outside of the tunnel. Reacting to this, an electronic climate control system operated fans that generated opposing warmed and ambient air flows to establish and maintain a temperature gradient from ambient at one end of the tunnel to approximately ambient +4 °C at the other end (Wurr et al., 1996). Air and soil temperatures were monitored continuously along the tunnel. Realistic seasonal and diurnal temperature fluctuations were therefore maintained within the tunnel, but with varying degrees of simulated climate warming depending on the position that seeds were placed along the tunnel.

A projected median emissions scenario for the local experimental area used in this work (West Midlands, UK) indicates an increase in the summer mean temperature of 3.7 °C by 2080 (UK Climate Change Projections, 2014; http://ukclimateprojections.metoffice.gov.uk/). We therefore adjusted the tunnel to a gradient from ambient to approximately + 4 °C this gave a soil temperature gradient of 2.5°C.

Seedling emergence: F_9 seeds produced at 15°C were used. Seedling emergence was performed in pots constructed as follows. Two 9 cm square black pots (TEKU VQF/V; BHGS Ltd, Evesham, UK) were placed inside each other and cut 2cm below the lip of the pots (Fig. 1) . A piece of 125 μm nylon mesh (45% open mesh: Plastok, Birkenhead, UK) was placed between the outer and inner pot sections and the two halves stapled together.

Figure 1. *Construction of pots to provide uniform replicated conditions to record seedling emergence from soil. The pots were placed in contact with capillary matting and surrounded by sand to space pots within replicate trays. Regular watering avoided differential moisture stress.*

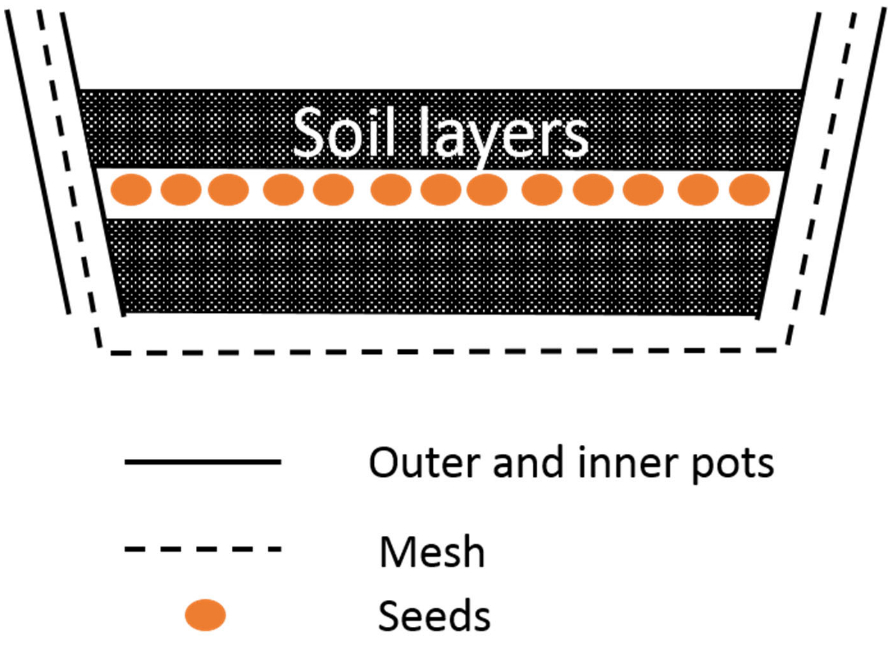

Into each pot was placed 50 mL of sterile soil, on top was dispersed 10 mg of seed which was covered with a further 75 mL of sterile soil. Individual RILS were chosen where the difference in flowering time between replicate plants was less than five days and the combined seed yield was greater than 200 mg. In this way 86 RILs (2 replicates each) were chosen that exhibited the full range of flowering times seen in the population. Seeds from the two replicate plants were sown in individual pots and placed in 1.2 metre square gravel trays (1.2 x 1.2 m x 12 cm deep; Garland Products Ltd, Kingswinford, UK) filled with sand as described below.

Trays were placed at three positions along the thermogradient tunnel being 1.45 m, 14.5 m and 28.5 m from the ambient end of the tunnel these positions were termed Ambient, Middle and Warm. The trays had drainage holes drilled in the base were lined with capillary matting and filled to a depth of 10 cm with horticultural grade sand which was covered with another layer of capillary matting. Pots were then placed on the capillary matting in a random block with a single replicate in each tray. Gaps between pots was then filled with sand and trays watered to saturation point using mist spray irrigation. Trays were set up at two times of year to simulate seed dispersal in the spring on 15-16 May 2013 (Winter annual behaviour) and in the autumn on 22-23 October 2013 (Summer annual behaviour). Soil in each pot was disturbed by stirring every 2 weeks and emerged seedlings recorded and removed. Pots were prevented from drying out by regular irrigation as above. Thermistore

temperature probes (Betatherm) linked to a data logger (Delta-T Devices) recorded soil temperature in each tray.

References

Alonso-Blanco C, Peeters AJ, Koornneef M, Lister C, Dean C, van den Bosch N, Pot J, Kuiper MT. 1998. Development of an AFLP based linkage map of Ler, Col and Cvi *Arabidopsis thaliana* ecotypes and construction of a Ler/Cvi recombinant inbred line population. *The Plant Journal* **14:** 259-271.

Bentsink L, Hanson J, Hanhart CJ, Blankestijn-de Vries H, Coltrane C, Keizer P, El-Lithy M, Alonso-Blanco C, de Andrés MT, Reymond M, et al. 2010. Natural variation for seed dormancy in *Arabidopsis* is regulated by additive genetic and molecular pathways. *Proceedings of the National Academy of Sciences USA* **107:** 4264-4269.

Broman, KW, Wu H, Sen Ś, Churchill GA. 2003. R/qtl: QTL mapping in experimental crosses. *Bioinformatics* **19:** 889–890.

Dekkers BJW, Bentsink L. 2015. Regulation of seed dormancy by abscisic acid and DELAY OF GERMINATION 1. *Seed Science Research* **25:** 82-98.

Footitt S, Douterelo-Soler I, Clay H, Finch-Savage WE. 2011. Dormancy cycling in *Arabidopsis* seeds is controlled by seasonally distinct hormone signalling pathways. *Proceedings of the National Academy of Sciences USA* **108:** 20236-20241

Footitt S, Finch-Savage WE. 2011. Production of seed samples for the effective molecular analysis of dormancy cycling in Arabidopsis. In: Kermode AR, ed. *Seed Dormancy*. London, UK: Humana Press 65-79.

Footitt S, Huang Z, Clay H, Mead A, Finch-Savage WE. 2013. Temperature, light and nitrate sensing coordinate *Arabidopsis* seed dormancy cycling resulting in winter and summer annual phenotypes. *The Plant Journal* **74:** 1003-1115

Joosen RV, Kodde J, Willems LA, Ligterink W, van der Plas LH, Hilhorst HW. 2010. GERMINATOR: a software package for high-throughput scoring and curve fitting of Arabidopsis seed germination. *The Plant Journal* **62:** 148-59.

Trapnell C, Roberts A, Goff L, Pertea G, Kim D, Kelley DR, Pimentel H, Salzberg SL, Rinn JL, Pachter L. 2012. Differential gene and transcript expression analysis of RNA-seq experiments with TopHat and Cufflinks. *Nature Protocols* **7:** 562–578.

Van Ooijen JW. 2009. MapQTL® 6, Software for the mapping of quantitative trait loci in experimental populations of diploid species. Kyazma B.V., Wageningen, Netherlands.

Voorrips RE. 2002. MapChart: software for the graphical presentation of linkage maps and QTLs. *Journal of Heredity* **93:** 77-78.

Walley PG, Carder J, Skipper E, Mathas E, Lynn J, Pink D, Buchanan-Wollaston V. 2012. A new framework broccoli x broccoli genetic map: better for breeders, better for complex trait analysis. *Theoretical and Applied Genetics* **124:** 467-484.

Wurr DCE, Fellows JR, Phelps K. 1996. Investigating trends in vegetable crop response to increasing temperature associated with climate change. *Scientia Horticulturae* **66:** 255-263.
